# Markers of Monocyte Activation, Inflammation, and Microbial Translocation Are Associated with Liver Fibrosis in Alcohol Use Disorder

**DOI:** 10.3390/jcm10163496

**Published:** 2021-08-08

**Authors:** Daniel Fuster, Xavier Garcia-Calvo, Oriol Farré, Paola Zuluaga, Ferran Bolao, Alba Leis, Anna Hernández-Rubio, Inmaculada Rivas, Robert Muga

**Affiliations:** 1 Addiction Unit, Department of Internal Medicine, Hospital Universitari Germans Trias i Pujol, 08916 Badalona, Barcelona, Spain; xavi_gc@msn.com (X.G.-C.); oriolfame@gmail.com (O.F.); ypzuluaga.germanstrias@gencat.cat (P.Z.); anna.hr92@gmail.com (A.H.-R.); rmuga.germanstrias@gencat.cat (R.M.); 2 Department of Medicine, Universitat Autònoma de Barcelona, 08916 Badalona, Barcelona, Spain; 3 Department of Internal Medicine, Hospital Universitari de Bellvitge, Institut d’Investigació Biomèdica de Bellvitge (IDIBELL), 08907 L’Hospitalet de Llobregat, Barcelona, Spain; fbolao@bellvitgehospital.cat; 4 Department of Biochemistry, Hospital Universitari Germans Trias i Pujol de Badalona, 08916 Badalona, Barcelona, Spain; aleis.germanstrias@gencat.cat; 5 Mental Health and Addiction Service, Badalona Serveis Assistencials-BSA, 08911 Badalona, Barcelona, Spain; irivas@bsa.cat

**Keywords:** alcohol use disorder, monocyte activation, inflammation, microbial translocation, liver fibrosis

## Abstract

Background: The association between markers of inflammation (interleukin (IL)-6 and IL-10), monocyte activation (sCD163 and sCD14), and microbial translocation (lipopolysaccharide (LPS) and LPS binding protein) and liver fibrosis in patients with alcohol use disorder (AUD) and no overt liver disease is not well established. Methods: We studied patients admitted for treatment of AUD at two hospitals in Barcelona. Advanced liver fibrosis (ALF) was defined as FIB-4 > 3.25. Results: A total of 353 participants (76.3% male) were included and 94 (26.5%) had ALF. In adjusted correlation analyses, sCD163, sCD14, IL-6, IL-10, and LPS binding protein levels directly correlated with FIB-4 values (adjusted correlation coefficients 0.214, 0.452, 0.317, 0.204, and 0.171, respectively). However, LPS levels were inversely associated with FIB-4 (−0.283). All plasma marker levels in the highest quartile, except LPS, were associated with ALF (sCD163, sCD14, IL-6, IL-10, and LPS binding protein: adjusted odds ratio (aOR) 11.49 (95% confidence interval 6.42–20.56), 1.87 (1.11–3.16), 2.99 (1.79–5.01), 1.84 (1.11–3.16), and 2.13 (1.30–3.50), respectively). Conversely, LPS levels in the lowest quartile were associated with ALF (aOR 2.58 (1.48–4.58), *p* < 0.01). Conclusion: In AUD patients, plasma levels of the markers of inflammation, monocyte activation, and microbial translocation are associated with ALF.

## 1. Introduction

Alcohol-related liver disease is the most frequent alcohol-related chronic medical problem and alcohol use is a major culprit of the global increase in liver-related deaths [1]. Alcohol use promotes microbial translocation due to changes in the microbiome and increased intestinal permeability, leading to systemic inflammation, monocyte activation, and progression of liver disease [2]. However, only a few of those who drink heavily eventually develop end-stage liver disease [3]. The early diagnosis of subjacent liver disease in otherwise healthy patients with AUD is intriguing [4] and there is an interest in obtaining markers to better stratify the risk of progressive liver injury among patients with unhealthy alcohol use [5].

All of the aforementioned pathological features (intestinal permeability, microbial translocation, systemic inflammation, and monocyte activation) have been thoroughly studied in patients with overt end-stage liver disease or severe alcoholic hepatitis [6,7,8,9], but they have been far less frequently assessed in patients with AUD without end-stage liver disease admitted for hospital treatment of the disorder [10,11].

Systemic inflammation can be measured via the plasma levels of interleukin (IL)-6 and IL-10. In addition, sCD14 and sCD163 are markers of monocyte activation [2], with sCD14 levels representing monocyte activation dependent on lipopolysaccharide (LPS). The binding of LPS to LPS binding protein (LBP) is a facilitator of the union of that complex to sCD14 and further activation of toll-like receptor 4 [2]. Increased levels of sCD163 are consistent with monocyte activation derived not only from the presence of LPS in peripheral blood but also from the presence of other damage-associated molecular patterns (DAMPs) that are produced by alcohol metabolism and increased iron deposition [11]. DAMPs trigger sterile inflammation and the activation of monocyte toll-like receptors, which subsequently activate quiescent stellate cells, leading to progressive liver fibrosis [12]. LPS and LBP levels are markers of microbial translocation that have been thoroughly studied in patients with cirrhosis of the liver [13] and in those with HIV infection with or without HCV co-infection [14,15]. However, they have been less frequently measured in patients admitted for the treatment of AUD [9,16].

Liver fibrosis is the main predictor of the progression to cirrhosis of the liver in patients with unhealthy alcohol use [17]. Despite liver biopsy being considered the gold standard for the evaluation of liver fibrosis, it is an invasive and costly procedure [18] and its performance is unlikely in patients with active alcohol or other drug use [19]. FIB-4 is a non-invasive index for estimating liver fibrosis that includes laboratory parameters that are usually monitored in everyday clinical practice [20]. It is a useful index for estimating liver fibrosis in patients with an alcohol or substance use disorder who rarely undergo a liver biopsy [19]. In addition to estimating the presence of liver fibrosis, FIB-4 also accurately predicts mortality and other poor health outcomes in other subsets of patients, such as incident heart failure [21].

The association between markers of inflammation and non-invasive measures of liver fibrosis has been previously studied [22], and markers of inflammation (e.g., IL-6) can predict the presence of advanced liver fibrosis (ALF) in HIV-infected patients [23]. However, whether markers of monocyte activation, intestinal permeability, and microbial translocation are associated with FIB-4 values consistent with ALF in patients with an AUD and no apparent end-stage liver disease remains to be elucidated.

We hypothesized that plasma levels of markers of monocyte activation (sCD163 and sCD14), inflammation (IL-6 and Il-10, and microbial translocation (lipopolysaccharide (LPS) and LPS binding protein (LBP)) are associated with FIB-4 levels in patients with AUD without overt end-stage liver disease admitted to hospital for the treatment of the disorder.

## 2. Materials and Methods

### 2.1. Participants

We included patients admitted for hospital alcohol detoxification at two tertiary teaching hospitals in Barcelona, Spain (Hospital Universitari Germans Trias i Pujol and Hospital Universitari de Bellvitge) between 2013 and 2019.

Patients were referred for hospital detoxification if they were deemed ineligible for ambulatory detoxification by addiction physicians. The main reasons for referring patients included a high risk of severe alcohol withdrawal, the presence of severe medical co-morbidities, and poor adherence to outpatient treatment and/or unsuccessful outpatient detoxification. Patients were excluded from the present study if they harbored autoimmune diseases, acute and decompensated medical co-morbidities, or severe mental health problems.

This study was approved by the Ethics Committee of both participant hospitals. Written consent was provided by all patients before study entry and participation. This study was conducted with compliance with ethical standards as well as the principles of good clinical practice defined by the World Medical Association’s Declaration of Helsinki [24].

### 2.2. Measurements

On the day of admission, all participants underwent a thorough physical examination and an interview regarding their history of alcohol consumption as well as their use of tobacco and other drugs. On the following day, blood was drawn to assess hematological and biochemical parameters and HCV infection. Additional information regarding the admission protocol and the methods for the detection of HCV infection as well as the measure of sCD163, sCD14, and IL-6 has been previously published [11,19,25].

Of note, the upper limit of detection for sCD163 was 1000 ng/mL and, for all assays above that threshold, a value of 1000 ng/m was set for the statistical analysis. IL-10 was determined by the same method as IL-6 [11] and the lower limit of detection was 0.02 pg/mL. For assays with an IL-10 concentration below the detection threshold, a value of 0.02 pg/mL was set for the analysis.

Plasma concentrations of LPS and LBP were determined by enzyme-linked immunosorbent assays. Each sample was diluted 10×. Plasma concentrations of LPS and LBP were measured in duplicate using the LPS ELISA kit (abx517692, Cambridge, UK) and the LBP ELISA kit (abx575210, Abbexa, Cambridge, UK), respectively. All assays were performed according to the manufacturer’s instructions. The inter-assay and intra-assay coefficients of variation were <10% for all analyses.

Liver fibrosis was assessed by the FIB-4 index [20]: age × AST (U/L)/platelet count (10^9^/L) × ALT (U/L)^1/2^. ALF was defined as FIB-4 > 3.25.

### 2.3. Statistical Analysis

Descriptive statistics were expressed as the median and interquartile range (IQR) for quantitative variables, or as absolute frequencies and percentages for qualitative variables. We performed the chi-squared test to explore significant differences in qualitative variables and the *t*-test for the mean differences in quantitative variables between participants that had ALF measured with FIB-4 and those who did not.

We performed an unadjusted correlation analysis between plasma marker levels and FIB-4 values as a continuous variable. In addition, we performed an adjusted correlation analysis where all correlations were adjusted for sex, alcohol intake, and the presence of HCV infection. We also performed logistic regression analyses to detect the association between plasma marker levels in the highest quartile and the presence of ALF measured with FIB-4. Finally, we performed a logistic regression analysis to detect the association between LPS levels in the lowest quartile and the presence of ALF. All logistic regression analyses were adjusted for sex, alcohol intake, and the presence HCV infection. The test results were considered significant if the resulting one-tailed *p*-value was < 0.05. Statistical analyses were performed using SPSS software version 15.0.1 (SPSS, Chicago, IL, USA).

## 3. Results

A total of 353 participants were included in the present study. Table 1 includes the baseline clinical characteristics and the median values for several laboratory parameters as well as for markers of monocyte activation (sCD163, sCD14), inflammation (IL-6 and IL-10), and microbial translocation (LPS and LBP).

Participants with ALF had a higher alcohol intake at admission (186 vs. 161 g/day, *p* < 0.05), a higher duration of AUD (22.8 vs. 17.5 years, *p* < 0.01), and a higher prevalence of HCV infection (23.6 vs. 9.3%, *p* < 0.01). As seen in Table 2, patients with ALF had higher mean sCD163 (944 vs. 638 ng/mL, *p* < 0.01), sCD14 (1.9 × 10^6^ vs. 1.7 × 10^6^ pg/mL, *p* < 0.01), LBP (55 vs. 35.4 pg/mL, *p* = 0.04), IL-6 (16.9 vs. 5.9 pg/mL, *p* = 0.04), and IL-10 (2.5 vs. 1.4 pg/mL, *p* < 0.01) levels and lower mean LPS values (1272 vs. 1975 pg/mL, *p* = 0.02).

**Figure 1:**
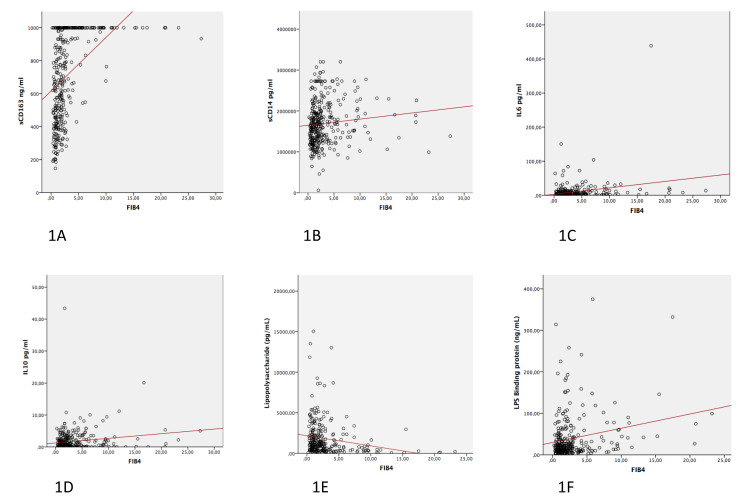
Unadjusted correlation plots between the different markers and FIB-4 levels: (**1A**) sCD163; (**1B**) sCD14; (**1C**) IL-6; (**1D**) IL-10; (**1E**) LPS; and (**1F**) LBP.

In addition, we performed correlation analyses between the different marker levels and FIB-4 levels as a continuous variable. The unadjusted correlation plots are seen in Figure 1.

Table 3 includes the results of the correlation analyses adjusted by sex, alcohol intake before admission, and the presence of HCV infection. As seen in Table 3, the levels of sCD163, sCD14, IL-6, IL-10, and LBP directly correlated with FIB-4 values (adjusted correlation coefficients 0.214, 0.452, 0.317, 0.204, and 0.171, respectively) whereas LPS levels were inversely associated with FIB-4 values (adjusted correlation coefficient −0.283).

Finally, we performed adjusted logistic regression analyses to assess the association between plasma marker levels in the highest quartile and the presence of ALF. All analyses were adjusted by sex, alcohol consumption, and the presence of HCV infection. As seen in Table 4, plasma levels of all markers, except LPS, in the highest quartile were significantly associated with the presence of ALF with an adjusted odds ratio ranging from 1.84 for IL-10 to 11.49 for sCD163. Conversely, LPS levels in the lowest quartile were significantly associated with the presence of ALF (adjusted odds ratio 2.58 (95% confidence interval: 1.48–4.58), *p* < 0.01).

## 4. Discussion

In this series of AUD patients with no decompensated liver disease admitted for hospital treatment of the disorder, plasma marker levels consistent with increased monocyte activation and increased systemic inflammation were associated with the presence of ALF, as were higher LBP levels.

In particular, levels of IL-6, IL-10, sCD14, sCD163, and LBP in the highest quartile and levels of LPS in the lowest quartile were associated with ALF measured with FIB-4. The same associations were found when marker levels were correlated with FIB-4 as a continuous variable; IL-10, sCD14, sCD163, and LBP levels directly correlated with FIB-4 values whereas the LPS levels negatively correlated with FIB-4. All analyses that detected these associations were adjusted by sex, amount of alcohol consumption before admission, and the presence of HCV infection. Notably, in this study population, patients with ALF had a greater alcohol consumption before admission and a higher prevalence of HCV infection.

As mentioned previously, LPS levels negatively correlated with FIB-4 values and LPS levels in the lowest quartile were associated with higher odds of ALF. These findings suggest that higher levels of LPS may be indicative of microbial translocation occurring in an earlier phase of the pathogenesis of alcohol-related liver disease when patients have not yet developed significant liver fibrosis. Monocyte activation, inflammation, and higher levels of LBP would be more prominent pathological features in later stages of the disease, which is why all those markers were associated with higher FIB-4 values and with higher odds of FIB-4 values suggestive of the presence of ALF.

The association between IL-6 levels and ALF has been previously described in a cohort of HIV-infected patients with unhealthy alcohol use [23] and in other cohorts of HIV-infected patients [26]. However, prior studies failed to detect an association between IL-10 levels and non-invasive markers of liver fibrosis [23]. The association between inflammatory markers and liver fibrosis is of interest as both higher IL-6 levels and FIB-4 values consistent with ALF have been associated with higher mortality and other health outcomes in different settings [27,28,29].

Other authors have shown that markers of monocyte activation, especially sCD14, have also been associated with mortality and other health complications [30,31]. In a study performed on HIV-infected patients, Sandler and colleagues showed that sCD14 levels correlated with AST and ferritin levels, which the authors found to be suggestive of liver inflammation [32]. In that same study, sCD4 levels correlated with gamma-glutamyl transpeptidase, alkaline phosphatase, and alpha-fetoprotein, which the authors found to be suggestive of liver fibrosis [32]. In a prior study by our group, sCD14 levels in the higher quartile were associated with AST levels whereas sCD163 values in the highest quartile were associated with AST and bilirubin levels and the presence of HCV infection, which suggests the presence of underlying a liver injury [11]. We are not aware of another study that has studied the association of plasma markers of monocyte activation and intestinal permeability and ALF in patients with AUD.

LPS levels have been studied in patients with AUD and seem to decrease with alcohol abstinence [9,33]. LBP levels are associated with mortality in patients with decompensated cirrhosis [34] and appear to decrease with the use of beta-blockers in patients with portal hypertension [13] but the levels in patients with AUD without overt liver disease have not received much attention in the literature. Our findings support the use of LBP as a surrogate marker of microbial translocation in patients with AUD. In addition, no other studies have evaluated the association between LPS and LBP levels and ALF.

Importantly, our findings underscore that monocyte activation, systemic inflammation, and microbial translocation are present in patients with AUD but without end-stage liver disease and admitted for hospital treatment, and that these levels are associated with FIB-4 values and the presence of ALF assessed with non-invasive measures. Patients with FIB-4 values suggestive of ALF should be prioritized to receive more intensive forms of follow-up treatment to secure alcohol abstinence. Abstinence from alcohol is associated with increased survival even in patients who already present with advanced forms of alcohol-related liver disease [3].

This study has a few limitations to be noted. First, plasma markers were assessed on the second day of hospital admission and their levels may be modified by alcohol abstinence. A sequential measurement of these markers during hospital alcohol detoxification is a potential future line of inquiry. Second, we included patients with the most severe forms of AUD (i.e., a 20-year history of the disorder and a median daily alcohol intake upon admission of 140 g/day) and our findings may not be extrapolated to patients with milder forms of unhealthy alcohol use as seen in primary care or hepatology clinics. Third, we estimated liver fibrosis with FIB-4, which has not been validated against the gold standard of liver biopsy for alcohol-related liver disease [35]. Despite that few authors have expressed concerns about the accuracy of FIB-4 [36], the European Association for the Study of the Liver has recently published an updated version of their guideline for the non-invasive diagnosis of a liver injury and recommends the use of FIB-4 as a first-line method for detecting patients with high probability of presenting significant liver disease [37]. The present study adds to the literature that categorizes FIB-4 as a reliable tool for estimating liver fibrosis in patients in whom the performance of a liver biopsy is unlikely [38]. In addition, FIB-4 includes variables that are routinely assessed in the everyday care of subjects with liver disease and is an ideal method of estimating liver fibrosis in resource-limited settings where the use of transient elastography is prohibitive. In addition to being a reliable predictor of mortality [29], FIB-4 values are associated with important disease correlates in this case series of patients with an AUD admitted for hospital treatment for the disorder.

In summary, the present study shows that higher levels of markers of inflammation, monocyte activation, and LBP and lower levels of LPS are associated with FIB-4 values as well as with ALF in patients admitted for the treatment of AUD.

## Figures and Tables

**Table 1:** Characteristics of the patients with alcohol use disorder admitted for hospital treatment.

Characteristic	*n* = 353
Men	271 (76.7)
Age (years)	50 (43–57)
BMI (kg/m^2^)	25.6 (22.6–29.2)
Alcohol consumption (g/day)	140 (100–224)
Duration of AUD (years)	20 (10–25)
HCV infection	44 (12.5)
Glucose (mg/dL)	92 (84.3–102.8)
Bilirubin (mg/dL)	0.67 (0.47–1.11)
AST (U/L)	39 (23–75)
ALT (U/L)	29 (18–50)
GGT (U/L)	93 (37–288.5)
Alkaline phosphatase (U/L)	79 (59–106.5)
Creatinine (mg/dL)	0.77 (0.65–0.89)
C-reactive protein (mg/L)	2.5 (0.9–5.9)
Leukocyte count (10^9^/L)	6.4 (5.0–7.6)
Monocyte count (10^9^/L)	0.60 (4.85–7.75)
Hemoglobin (g/dL)	14 (12.7–15.2)
Median corpuscular volume (fl)	94.7 (90.9–99)
Platelet count (10^9^/L)	191 (137–242)
Fibrinogen (mg/dL)	329 (269–388)
Erythrocyte sedimentation rate (mm)	12 (5–27)
Ferritin (ng/mL)	185.75 (81.25–386.83)
FIB-4	1.92 (1.07–3.69)
Advanced liver fibrosis (FIB-4 > 3.25)	94 (27.4)
sCD163 (ng/mL)	763 (476–1000)
sCD14 (×10^6^ pg/mL)	1.65 (1.29–1.97)
IL-6 (pg/mL)	3.31 (0.87–8.16)
IL-10 (pg/mL)	0.63 (0.02–2.30)
LPS (pg/mL)	1075 (410–2282)
LBP (pg/mL)	20 (10.5–44.8)

Values are given as *n* (%) or median (IQR). HCV: hepatitis C virus; LPS: lipopolysaccharide; LBP: lipopolysaccharide binding protein.

**Table 2:** Mean plasma marker values stratified by the presence of advanced liver fibrosis.

	No ALF	ALF	*p*-Value
sCD163 (ng/mL)	638 (256)	944 (130)	<0.01
sCD14 (×10^6^ pg/mL)	1.7 (0.5)	1.9 (0.5)	<0.01
IL-6 (pg/mL)	5.9 (13.9)	16.9 (48.0)	0.04
IL-10 (pg/mL)	1.4 (3.2)	2.5 (3.3)	<0.01
LPS (pg/mL)	1975 (2217)	1272 (2071)	0.02
LBP (pg/mL)	35.4 (46.0)	55 (71.3)	<0.01

LPS: lipopolysaccharide; LBP: lipopolysaccharide binding protein; ALF: advanced liver fibrosis.

**Table 3:** Adjusted correlations between plasma marker levels and FIB-4 values.

	Adjusted Correlation Coefficient ^a^	*p*-Value
sCD163	0.214	<0.01
sCD14	0.452	<0.01
IL-6	0.317	<0.01
IL-10	0.204	<0.01
LPS	−0.283	<0.01
LBP	0.171	<0.01

^a^ Adjusted for sex, alcohol intake (g/day), and HCV infection. LBP: lipopolysaccharide binding protein. Each row represents a different model.

**Table 4:** Logistic regression for the association between marker levels in the highest quartile and advanced liver fibrosis.

	Adjusted Odds Ratio (95% Confidence Interval) ^a^	*p*-Value
sCD163	11.49 (6.42–20.56)	<0.01
sCD14	1.87 (1.11–3.16)	0.02
IL-6	2.99 (1.79–5.01)	<0.01
IL-10	1.84 (1.11–3.16)	0.02
LPS	1.09 (0.66–1.79)	0.74
LBP	2.13 (1.30–3.50)	<0.01

The highest quartile was compared with all other values. Each row represents a different model. ^a^ Adjusted for sex, alcohol intake, and HCV infection.

## Data Availability

The raw data presented in this study are available to any scientist wishing to use them for non-commercial purposes on request from the corresponding author without breaching participant confidentiality. The data are not publicly available due to privacy.
